# Supplementary material for: Cell-specific regulation of gene expression using splicing-dependent frameshifting
Source: Nat Commun. 2022 Oct 1;13:5773. doi: 10.1038/s41467-022-33523-2 (PMC9526712; doi:10.1038/s41467-022-33523-2)
Supplement: Supplementary file 2 — Description of additional Supplementary File [file 41467_2022_33523_MOESM2_ESM.pdf]

## **Descriptions of additional Supplementary files**

Supplementary Video 1. CaRPv1_10x.mp4 Video of primary rat neuronal cultures transfected with SLED.CaRPv1 and imaged using a 10x objective at 2Hz (framerate = 7fps). Images were normalized and processed according to the protocol listed in the Methods section.

Supplementary Video 2. CaRPv1_20x.mp4 Video of primary rat neuronal cultures transfected with SLED.CaRPv1 (bicuculline treated) and imaged using a 20x objective at 4Hz (framerate = 30fps). Although transfection of primary rat cultures leads is sparse, occasionally transfected neurons are close enough in proximity for simultaneous imaging at 20x magnification. Images were normalized and processed according to the protocol listed in the Methods section.
