# Supplementary material for: Cell-specific regulation of gene expression using splicing-dependent frameshifting
Source: Nat Commun. 2022 Oct 1;13:5773. doi: 10.1038/s41467-022-33523-2 (PMC9526712; doi:10.1038/s41467-022-33523-2)
Supplement: Supplementary file 5 — Reporting Summary [file 41467_2022_33523_MOESM5_ESM.pdf]

nature portfolio

Corresponding author(s): Jonathan Ling and Seth Blackshaw

Last updated by author(s): Jun 29, 2022

Reporting Summary

Nature Portfolio wishes to improve the reproducibility of the work that we publish. This form provides structure for consistency and transparency in reporting. For further information on Nature Portfolio policies, see our Editorial Policies and the Editorial Policy Checklist.

Statistics

For all statistical analyses, confirm that the following items are present in the figure legend, table legend, main text, or Methods section.

n/a	Confirmed	
☐	☒	The exact sample size (*n*) for each experimental group/condition, given as a discrete number and unit of measurement
☐	☒	A statement on whether measurements were taken from distinct samples or whether the same sample was measured repeatedly
☐	☒	The statistical test(s) used AND whether they are one- or two-sided<br>*Only common tests should be described solely by name; describe more complex techniques in the Methods section.*
☐	☒	A description of all covariates tested
☐	☒	A description of any assumptions or corrections, such as tests of normality and adjustment for multiple comparisons
☐	☒	A full description of the statistical parameters including central tendency (e.g. means) or other basic estimates (e.g. regression coefficient) AND variation (e.g. standard deviation) or associated estimates of uncertainty (e.g. confidence intervals)
☐	☒	For null hypothesis testing, the test statistic (e.g. *F*, *t*, *r*) with confidence intervals, effect sizes, degrees of freedom and *P* value noted<br>*Give P values as exact values whenever suitable.*
☒	☐	For Bayesian analysis, information on the choice of priors and Markov chain Monte Carlo settings
☒	☐	For hierarchical and complex designs, identification of the appropriate level for tests and full reporting of outcomes
☒	☐	Estimates of effect sizes (e.g. Cohen's *d*, Pearson's *r*), indicating how they were calculated

Our web collection on statistics for biologists contains articles on many of the points above.

Software and code

Policy information about availability of computer code

Data collection	Zeiss imaging software, ZEN v3.4
Data analysis	All splicing analysis scripts used are available at the repository: https://github.com/jpling/ascot<br>Microsoft Excel 365, Imaris 9.9 and GraphPad Prism 9 were also used for data analysis

For manuscripts utilizing custom algorithms or software that are central to the research but not yet described in published literature, software must be made available to editors and reviewers. We strongly encourage code deposition in a community repository (e.g. GitHub). See the Nature Portfolio guidelines for submitting code & software for further information.

Data

Policy information about availability of data

All manuscripts must include a data availability statement. This statement should provide the following information, where applicable:

- Accession codes, unique identifiers, or web links for publicly available datasets
- A description of any restrictions on data availability
- For clinical datasets or third party data, please ensure that the statement adheres to our policy

Public RNA-Seq data was used in this study, no RNA-Seq data was generated in this study

Field-specific reporting

Please select the one below that is the best fit for your research. If you are not sure, read the appropriate sections before making your selection.

☒ Life sciences ☐ Behavioural & social sciences ☐ Ecological, evolutionary & environmental sciences

For a reference copy of the document with all sections, see nature.com/documents/nr-reporting-summary-flat.pdf

Life sciences study design

All studies must disclose on these points even when the disclosure is negative.

Sample size	Sample sizes were chosen with a minimum of n=3 if samples represent averages across large cell populations or tissues (e.g. retinas for subretinal injections or independent FACS experiments of thousands of cells). For SLED ratio quantifications from microscopy images, a minimum of n=50 cells were counted across sections, although more cells were counted if available in the field of view. No prior sample size calculation was performed before measurement. Our choice of sample size is adequate for this study given that the strong specificity of SLED vectors led to strong statistical validation.
Data exclusions	None
Replication	SLED vectors were repeated across multiple species to test whether specificity could be replicated in different organisms. No variation in specificity was observed between multiple injections or transfections for a given experimental condition. Experiments were performed at least n=3 times across multiple in vitro transfections or multiple AAV injections of SLED vectors. Independent replicates were successful except in cases of technical error (e.g. mistargeted surgical injections, loss of cell culture viability, etc). In these cases, experiments were again performed once technical errors were corrected.
Randomization	Subretinal injections of SLED vectors were performed in a blinded and randomized manner. Analyses of ERG and OCT data were analyzed in a double-blinded manner. Randomization was not necessary for testing SLED vectors, given that randomization is inherent to the tissues tested. Targeted cell types and off-target cell types exist in the same tissue sample and are both transduced by AAV, thus providing a randomized internal control.
Blinding	The identity of the specific viruses used for subretinal injections were blind to the experimenter performing injections by another member of the lab. Mice only labeled by a generic identifier were then sent to CCF for ERG analysis. Data was unblinded after ERG analysis was complete and results were shared (prior to unblinding).

Behavioural & social sciences study design

All studies must disclose on these points even when the disclosure is negative.

Study description	*Briefly describe the study type including whether data are quantitative, qualitative, or mixed-methods (e.g. qualitative cross-sectional, quantitative experimental, mixed-methods case study).*
Research sample	*State the research sample (e.g. Harvard university undergraduates, villagers in rural India) and provide relevant demographic information (e.g. age, sex) and indicate whether the sample is representative. Provide a rationale for the study sample chosen. For studies involving existing datasets, please describe the dataset and source.*
Sampling strategy	*Describe the sampling procedure (e.g. random, snowball, stratified, convenience). Describe the statistical methods that were used to predetermine sample size OR if no sample-size calculation was performed, describe how sample sizes were chosen and provide a rationale for why these sample sizes are sufficient. For qualitative data, please indicate whether data saturation was considered, and what criteria were used to decide that no further sampling was needed.*
Data collection	*Provide details about the data collection procedure, including the instruments or devices used to record the data (e.g. pen and paper, computer, eye tracker, video or audio equipment) whether anyone was present besides the participant(s) and the researcher, and whether the researcher was blind to experimental condition and/or the study hypothesis during data collection.*
Timing	*Indicate the start and stop dates of data collection. If there is a gap between collection periods, state the dates for each sample cohort.*
Data exclusions	*If no data were excluded from the analyses, state so OR if data were excluded, provide the exact number of exclusions and the rationale behind them, indicating whether exclusion criteria were pre-established.*
Non-participation	*State how many participants dropped out/declined participation and the reason(s) given OR provide response rate OR state that no participants dropped out/declined participation.*
Randomization	*If participants were not allocated into experimental groups, state so OR describe how participants were allocated to groups, and if allocation was not random, describe how covariates were controlled.*

Ecological, evolutionary & environmental sciences study design

All studies must disclose on these points even when the disclosure is negative.

Study description	*Briefly describe the study. For quantitative data include treatment factors and interactions, design structure (e.g. factorial, nested, hierarchical), nature and number of experimental units and replicates.*
Research sample	*Describe the research sample (e.g. a group of tagged Passer domesticus, all Stenocereus thurberi within Organ Pipe Cactus National Monument), and provide a rationale for the sample choice. When relevant, describe the organism taxa, source, sex, age range and any manipulations. State what population the sample is meant to represent when applicable. For studies involving existing datasets, describe the data and its source.*
Sampling strategy	*Note the sampling procedure. Describe the statistical methods that were used to predetermine sample size OR if no sample-size calculation was performed, describe how sample sizes were chosen and provide a rationale for why these sample sizes are sufficient.*
Data collection	*Describe the data collection procedure, including who recorded the data and how.*
Timing and spatial scale	*Indicate the start and stop dates of data collection, noting the frequency and periodicity of sampling and providing a rationale for these choices. If there is a gap between collection periods, state the dates for each sample cohort. Specify the spatial scale from which the data are taken*
Data exclusions	*If no data were excluded from the analyses, state so OR if data were excluded, describe the exclusions and the rationale behind them, indicating whether exclusion criteria were pre-established.*
Reproducibility	*Describe the measures taken to verify the reproducibility of experimental findings. For each experiment, note whether any attempts to repeat the experiment failed OR state that all attempts to repeat the experiment were successful.*
Randomization	*Describe how samples/organisms/participants were allocated into groups. If allocation was not random, describe how covariates were controlled. If this is not relevant to your study, explain why.*
Blinding	*Describe the extent of blinding used during data acquisition and analysis. If blinding was not possible, describe why OR explain why blinding was not relevant to your study.*

Did the study involve field work? ☐ Yes ☐ No

Field work, collection and transport

Field conditions	*Describe the study conditions for field work, providing relevant parameters (e.g. temperature, rainfall).*
Location	*State the location of the sampling or experiment, providing relevant parameters (e.g. latitude and longitude, elevation, water depth).*
Access & import/export	*Describe the efforts you have made to access habitats and to collect and import/export your samples in a responsible manner and in compliance with local, national and international laws, noting any permits that were obtained (give the name of the issuing authority, the date of issue, and any identifying information).*
Disturbance	*Describe any disturbance caused by the study and how it was minimized.*

Reporting for specific materials, systems and methods

We require information from authors about some types of materials, experimental systems and methods used in many studies. Here, indicate whether each material, system or method listed is relevant to your study. If you are not sure if a list item applies to your research, read the appropriate section before selecting a response.

Materials & experimental systems

n/a	Involved in the study	
☐	☒	Antibodies
☐	☒	Eukaryotic cell lines
☒	☐	Palaeontology and archaeology
☐	☒	Animals and other organisms
☒	☐	Human research participants
☒	☐	Clinical data
☒	☐	Dual use research of concern

Methods

n/a	Involved in the study	
☒	☐	ChIP-seq
☐	☒	Flow cytometry
☒	☐	MRI-based neuroimaging

Antibodies

Antibodies used	The following antibodies were used for primary culture neurons (supplier, catalog number and working dilution are indicated): anti-Gad67 ms IgG2a (Millipore MAB5406, 1:500); anti-somatostatin rat (Millipore MAB354, 1:400); anti-HuCD IgG2B (Thermo 16A11, 1:200); anti-NeuN mouse IgG1 (Thermo MAB377, 1:500). The following antibodies were used for brain sections (supplier, catalog number and working dilution are indicated): anti-GFP chicken polyclonal (Abcam ab13970, 1:2000); anti-dsRED rabbit polyclonal (Tanaka LivingColors 632496, 1:1000) (this antibody also detects mCherry); anti-NeuN mouse monoclonal IgG1 (Thermo MAB377, 1:500); anti-Gad67 mouse monoclonal IgG2a (Millipore MAB5406, 1:200); anti-PV mouse monoclonal IgG1 (Swant PV235, 1:2000); anti-somatostatin rat monoclonal IgG2b (Millipore MAB354, 1:400).
Validation	Manufacturer notes on validation:<br>Anti-Gad67 (Millipore MAB5406): Reacts with the 67kDa isoform of Glutamate Decarboxylase (GAD67) of rat, mouse and human origins, other species not yet tested. No detectable cross reactivity with GAD65 by Western blot on rat brain lysate when compared to blot probed with AB1511 that reacts with both GAD65 & GAD67.<br>Anti-somatostatin (Millipore MAB354): Recognizes Somatostatin. Shows no cross-reactivity to enkephalins, other endorphins, substance P or CGRP. Partially cross-reacts with somatostatin fragments.<br>Anti-HuCD (Thermo 16A11): This antibody recognizes the Elav family members HuC, HuD and Hel-N1 neuronal proteins. It does not recognize HuR, another Elav family member that is present in all proliferating cells. The antibody has been shown to specifically label neuronal cells in zebrafish, chick, canaries, and humans, and is likely to label neuronal cells in most vertebrate species. Labeling is visible early in development, at about the time that the neurons leave the mitotic cycle.<br>Anti-NeuN (Thermo MAB377): MILLIPORE's exclusive monoclonal antibody to vertebrate neuron-specific nuclear protein called NeuN (or Neuronal Nuclei) reacts with most neuronal cell types throughout the nervous system of mice including cerebellum, cerebral cortex, hippocampus, thalamus, spinal cord and neurons in the peripheral nervous system including dorsal root ganglia, sympathetic chain ganglia and enteric ganglia. Developmentally, immunoreactivity is first observed shortly after neurons have become postmitotic, no staining has been observed in proliferative zones. The immunohistochemical staining is primarily localized in the nucleus of the neurons with lighter staining in the cytoplasm. The few cell types not reactive with MAB377 include Purkinje, mitral and photoreceptor cells. The antibody is an excellent marker for neurons in primary cultures and in retinoic acid-stimulated P19 cells. It is also useful for identifying neurons in transplants.<br>Anti-GFP (Abcam ab13970): Our GFP antibody does cross-react with the many fluorescent proteins that are derived from the jellyfish Aequorea victoria. These are all proteins that differ from the original GFP by just a few point mutations (EGFP, YFP, mVenus, CFP, BFP etc.).<br>Anti-dsRED (Tanaka LivingColors 632496): The Living Colors DsRed Polyclonal Antibody has been raised against DsRed-Express, a variant of Discosoma sp. red fluorescent protein. This antibody recognizes DsRed-Express, DsRed-Express2, mCherry, DsRed2, E2-Crimson, tdTomato, mStrawberry, and mBanana, and both N- and C-terminal fusion proteins containing these fluorescent proteins in mammalian cell lysates.<br>Anti-PV (Swant PV235): McAB 235 against parvalbumin specifically localizes parvalbumin using free-floating or mounted sections of brain and muscles probably of all vertebrates. Fixation does not seem to be a hindrance to the detection of the antigen since even 2.5% glutaraldehyde was compatible with immunolocalization on semithin (0.5 µm thin) cryo-sections of the rat cortex. It can be used with rat-parvalbumin as a tracer to quantify parvalbumin in the brain of various species.

Eukaryotic cell lines

Policy information about cell lines

Cell line source(s)	HEK293 (CRL-1573), HepG2 (HB-8065), N2a (CCL-131), and MP41 (CRL-3297) cells were obtained from ATCC. Mel-202 (13012457) cells were obtained from MilliporeSigma. K562 cells and derivates were obtained from Horizon Discovery. 92-1 uveal melanoma cell lines were obtained from the laboratory of Dr. Charles Eberhart (Johns Hopkins School of Medicine).
Authentication	Cells used for SF3B1 studies were tested for mutation status. SF3B1 genotype was confirmed in all lines by Sanger sequencing of exons 13–24. STR cell line authentication and mycoplasma testing was performed prior to the beginning of studies. All other cells were not authenticated.
Mycoplasma contamination	Cell lines were not tested for mycoplasma contamination
Commonly misidentified lines<br>(See ICLAC register)	No commonly misidentified lines were used

Palaeontology and Archaeology

Specimen provenance	*Provide provenance information for specimens and describe permits that were obtained for the work (including the name of the issuing authority, the date of issue, and any identifying information). Permits should encompass collection and, where applicable, export.*
Specimen deposition	*Indicate where the specimens have been deposited to permit free access by other researchers.*
Dating methods	*If new dates are provided, describe how they were obtained (e.g. collection, storage, sample pretreatment and measurement), where they were obtained (i.e. lab name), the calibration program and the protocol for quality assurance OR state that no new dates are provided.*

☐ Tick this box to confirm that the raw and calibrated dates are available in the paper or in Supplementary Information.

Ethics oversight | *Identify the organization(s) that approved or provided guidance on the study protocol, OR state that no ethical approval or guidance was required and explain why not.*

Note that full information on the approval of the study protocol must also be provided in the manuscript.

Animals and other organisms

Policy information about studies involving animals; ARRIVE guidelines recommended for reporting animal research

Laboratory animals	Male and female C57BL/6J mice and rds mice were obtained from The Jackson Laboratory, as indicated in materials and methods. Two month old C57BL/6J mice were used for testing SLED vectors in normal tissue. P0 Rds mice were used for intravitreal retina injections of PRPH2 expressing vectors and aged to 3-4 months before OCT and ERG analysis. All mice were housed in 14-hour light/10-hour dark cycles in ambient temperature and humidity.
Wild animals	No wild animals were used
Field-collected samples	No field-collected samples were used
Ethics oversight	All animals were treated in accordance with the Johns Hopkins University Animal Care and Use Committee (IACUC) guidelines, protocl MO22M22

Note that full information on the approval of the study protocol must also be provided in the manuscript.

Human research participants

Policy information about studies involving human research participants

Population characteristics	*Describe the covariate-relevant population characteristics of the human research participants (e.g. age, gender, genotypic information, past and current diagnosis and treatment categories). If you filled out the behavioural & social sciences study design questions and have nothing to add here, write "See above."*
Recruitment	*Describe how participants were recruited. Outline any potential self-selection bias or other biases that may be present and how these are likely to impact results.*
Ethics oversight	*Identify the organization(s) that approved the study protocol.*

Note that full information on the approval of the study protocol must also be provided in the manuscript.

Clinical data

Policy information about clinical studies

All manuscripts should comply with the ICMJEguidelines for publication of clinical research and a completedCONSORT checklist must be included with all submissions.

Clinical trial registration	*Provide the trial registration number from ClinicalTrials.gov or an equivalent agency.*
Study protocol	*Note where the full trial protocol can be accessed OR if not available, explain why.*
Data collection	*Describe the settings and locales of data collection, noting the time periods of recruitment and data collection.*
Outcomes	*Describe how you pre-defined primary and secondary outcome measures and how you assessed these measures.*

Dual use research of concern

Policy information about dual use research of concern

Hazards

Could the accidental, deliberate or reckless misuse of agents or technologies generated in the work, or the application of information presented in the manuscript, pose a threat to:

No	Yes	
☐	☐	Public health
☐	☐	National security
☐	☐	Crops and/or livestock
☐	☐	Ecosystems
☐	☐	Any other significant area

Experiments of concern

Does the work involve any of these experiments of concern:

No	Yes	
☐	☐	Demonstrate how to render a vaccine ineffective
☐	☐	Confer resistance to therapeutically useful antibiotics or antiviral agents
☐	☐	Enhance the virulence of a pathogen or render a nonpathogen virulent
☐	☐	Increase transmissibility of a pathogen
☐	☐	Alter the host range of a pathogen
☐	☐	Enable evasion of diagnostic/detection modalities
☐	☐	Enable the weaponization of a biological agent or toxin
☐	☐	Any other potentially harmful combination of experiments and agents

ChIP-seq

Data deposition

☐ Confirm that both raw and final processed data have been deposited in a public database such as GEO.

☐ Confirm that you have deposited or provided access to graph files (e.g. BED files) for the called peaks.

Data access links<br>*May remain private before publication.*	*For "Initial submission" or "Revised version" documents, provide reviewer access links. For your "Final submission" document, provide a link to the deposited data.*
Files in database submission	*Provide a list of all files available in the database submission.*
Genome browser session<br>(e.g. UCSC)	*Provide a link to an anonymized genome browser session for "Initial submission" and "Revised version" documents only, to enable peer review. Write "no longer applicable" for "Final submission" documents.*

Methodology

Replicates	*Describe the experimental replicates, specifying number, type and replicate agreement.*
Sequencing depth	*Describe the sequencing depth for each experiment, providing the total number of reads, uniquely mapped reads, length of reads and whether they were paired- or single-end.*
Antibodies	*Describe the antibodies used for the ChIP-seq experiments; as applicable, provide supplier name, catalog number, clone name, and lot number.*
Peak calling parameters	*Specify the command line program and parameters used for read mapping and peak calling, including the ChIP, control and index files used.*
Data quality	*Describe the methods used to ensure data quality in full detail, including how many peaks are at FDR 5% and above 5-fold enrichment.*
Software	*Describe the software used to collect and analyze the ChIP-seq data. For custom code that has been deposited into a community repository, provide accession details.*

Flow Cytometry

Plots

Confirm that:

☒ The axis labels state the marker and fluorochrome used (e.g. CD4-FITC).

☒ The axis scales are clearly visible. Include numbers along axes only for bottom left plot of group (a 'group' is an analysis of identical markers).

☒ All plots are contour plots with outliers or pseudocolor plots.

☒ A numerical value for number of cells or percentage (with statistics) is provided.

Methodology

Sample preparation	After transfection or AAV transduction of SLED vectors, cells were processed into single cell suspensions ready for FACS
Instrument	BD FACSAria

Software	FlowJo
Cell population abundance	Sorted cell populations were isolated in high enough abundance to obtain total RNA from cells. Sample quality was validated using RT-PCR post-FACS.
Gating strategy	Cells were enriched for the flip variant by sorting a high EGFP and low mCherry cell population

☒ Tick this box to confirm that a figure exemplifying the gating strategy is provided in the Supplementary Information.

Magnetic resonance imaging

Experimental design

Design type	*Indicate task or resting state; event-related or block design.*
Design specifications	*Specify the number of blocks, trials or experimental units per session and/or subject, and specify the length of each trial or block (if trials are blocked) and interval between trials.*
Behavioral performance measures	*State number and/or type of variables recorded (e.g. correct button press, response time) and what statistics were used to establish that the subjects were performing the task as expected (e.g. mean, range, and/or standard deviation across subjects).*

Acquisition

Imaging type(s)	*Specify: functional, structural, diffusion, perfusion.*
Field strength	*Specify in Tesla*
Sequence & imaging parameters	*Specify the pulse sequence type (gradient echo, spin echo, etc.), imaging type (EPI, spiral, etc.), field of view, matrix size, slice thickness, orientation and TE/TR/flip angle.*
Area of acquisition	*State whether a whole brain scan was used OR define the area of acquisition, describing how the region was determined.*

Diffusion MRI ☐ Used ☐ Not used

Preprocessing

Preprocessing software	*Provide detail on software version and revision number and on specific parameters (model/functions, brain extraction, segmentation, smoothing kernel size, etc.).*
Normalization	*If data were normalized/standardized, describe the approach(es): specify linear or non-linear and define image types used for transformation OR indicate that data were not normalized and explain rationale for lack of normalization.*
Normalization template	*Describe the template used for normalization/transformation, specifying subject space or group standardized space (e.g. original Talairach, MNI305, ICBM152) OR indicate that the data were not normalized.*
Noise and artifact removal	*Describe your procedure(s) for artifact and structured noise removal, specifying motion parameters, tissue signals and physiological signals (heart rate, respiration).*
Volume censoring	*Define your software and/or method and criteria for volume censoring, and state the extent of such censoring.*

Statistical modeling & inference

Model type and settings	*Specify type (mass univariate, multivariate, RSA, predictive, etc.) and describe essential details of the model at the first and second levels (e.g. fixed, random or mixed effects; drift or auto-correlation).*
Effect(s) tested	*Define precise effect in terms of the task or stimulus conditions instead of psychological concepts and indicate whether ANOVA or factorial designs were used.*

Specify type of analysis: ☐ Whole brain ☐ ROI-based ☐ Both

Statistic type for inference (See Eklund et al. 2016)	*Specify voxel-wise or cluster-wise and report all relevant parameters for cluster-wise methods.*
Correction	*Describe the type of correction and how it is obtained for multiple comparisons (e.g. FWE, FDR, permutation or Monte Carlo).*

Models & analysis

n/a | Involved in the study

- [ ] [ ] Functional and/or effective connectivity
- [ ] [ ] Graph analysis
- [ ] [ ] Multivariate modeling or predictive analysis

Functional and/or effective connectivity	*Report the measures of dependence used and the model details (e.g. Pearson correlation, partial correlation, mutual information).*
Graph analysis	*Report the dependent variable and connectivity measure, specifying weighted graph or binarized graph, subject- or group-level, and the global and/or node summaries used (e.g. clustering coefficient, efficiency, etc.).*
Multivariate modeling and predictive analysis	*Specify independent variables, features extraction and dimension reduction, model, training and evaluation metrics.*
